# Evaluation of Nine Consensus Indices in Delphi Foresight Research and Their Dependency on Delphi Survey Characteristics: A Simulation Study and Debate on Delphi Design and Interpretation

**DOI:** 10.1371/journal.pone.0135162

**Published:** 2015-08-13

**Authors:** Stanislav Birko, Edward S. Dove, Vural Özdemir

**Affiliations:** 1 Centre of Genomics and Policy, Department of Human Genetics, Faculty of Medicine, McGill University, Montreal, QC, Canada; 2 J. Kenyon Mason Institute for Medicine, Life Sciences and the Law, University of Edinburgh School of Law, Edinburgh, United Kingdom; 3 Faculty of Communications, Office of the President, International Technology and Innovation Policy, Gaziantep University, Üniversite Bulvarı, Şehitkamil, Gaziantep, 27310, Turkey; 4 Department of Industrial Engineering, Faculty of Engineering, Gaziantep University, Üniversite Bulvarı, Şehitkamil, Gaziantep, 27310, Turkey; 5 Amrita School of Biotechnology, Amrita Vishwa Vidyapeetham, Amrita University, Amritapuri, Clappana P.O., Kollam, Kerala, India; Örebro University, SWEDEN

## Abstract

The extent of consensus (or the lack thereof) among experts in emerging fields of innovation can serve as antecedents of scientific, societal, investor and stakeholder synergy or conflict. Naturally, how we measure consensus is of great importance to science and technology strategic foresight. The Delphi methodology is a widely used anonymous survey technique to evaluate consensus among a panel of experts. Surprisingly, there is little guidance on how indices of consensus can be influenced by parameters of the Delphi survey itself. We simulated a classic three-round Delphi survey building on the concept of clustered consensus/dissensus. We evaluated three study characteristics that are pertinent for design of Delphi foresight research: (1) the number of survey questions, (2) the sample size, and (3) the extent to which experts conform to group opinion (the Group Conformity Index) in a Delphi study. Their impacts on the following nine Delphi consensus indices were then examined in 1000 simulations: Clustered Mode, Clustered Pairwise Agreement, Conger’s Kappa, De Moivre index, Extremities Version of the Clustered Pairwise Agreement, Fleiss’ Kappa, Mode, the Interquartile Range and Pairwise Agreement. The dependency of a consensus index on the Delphi survey characteristics was expressed from 0.000 (no dependency) to 1.000 (full dependency). The number of questions (range: 6 to 40) in a survey did not have a notable impact whereby the dependency values remained below 0.030. The variation in sample size (range: 6 to 50) displayed the top three impacts for the Interquartile Range, the Clustered Mode and the Mode (dependency = 0.396, 0.130, 0.116, respectively). The Group Conformity Index, a construct akin to measuring stubbornness/flexibility of experts’ opinions, greatly impacted all nine Delphi consensus indices (dependency = 0.200 to 0.504), except the Extremity CPWA and the Interquartile Range that were impacted only beyond the first decimal point (dependency = 0.087 and 0.083, respectively). Scholars in technology design, foresight research and future(s) studies might consider these new findings in strategic planning of Delphi studies, for example, in rational choice of consensus indices and sample size, or accounting for confounding factors such as experts’ variable degrees of conformity (stubbornness/flexibility) in modifying their opinions.

## Introduction

The extent of consensus among experts in new fields of knowledge can serve as antecedents of scientific, societal, investor and stakeholder synergy and conflict, and by extension, help derive foresight on future innovation scenarios. Naturally, how we evaluate consensus (or the lack thereof) in a given field of science and technology matters to foresight research.

Delphi studies are a cornerstone in deciphering the emerging technology and innovation future(s), helping guide attendant public policies. The Delphi methodology is a widely used group survey technique, typically conducted over three consecutive rounds, to evaluate consensus among experts in a field. A Delphi study is conducted with a group of individuals considered to have expertise (both professional and experience-based) in the field under investigation. The survey rounds iteratively ask the experts to prioritize the issues or rate them on implementation-related scales such as feasibility or desirability, providing controlled feedback of the previous round’s group results [1, 2]. A moderating researcher oversees the Delphi survey, in the course of which the participants remain anonymous to each other, but not to the moderator [3]. By virtue of experts’ anonymity and the iterative group communication, the Delphi methodology is thought to be less subject to peer pressure and bias from experts with dominant personalities or to pressure from oneself to defend a previously stated opinion [4].

Sinha et al. has underscored that Delphi research is increasingly being used to gauge consensus around many topics in life sciences and medicine, such as education, development of clinical guidelines, and prioritisation of research topics [5]. Moreover, Delphi studies are important for technology foresight and for identifying the knowledge domains on which the innovation actors may have no consensus, and by extension, a “clean slate” to be receptive to new policy interventions for anticipatory governance of new technology and innovation:

When there is consensus on a given subject, it may mean the experts are already “entrenched” firmly in their opinions and are unlikely to change their stances easily; they might be resistant to guidance by new insights or innovation policies on that subject matter. Hence, issues where there is no consensus at all are worthy of careful reconsideration for future policy design because such topics without a consensus might actually be the real-life *actionable* target issues where “change is still possible” by new policies. [6]

As early as 1975, Adelson and Aroni found that Delphi surveys offered a valuable tool in elucidating strategic foresight on “emergence trajectories”, be they new technologies, innovative products or fields of knowledge:

[B]oth consensus and dissensus on images of the future [are] useful to understand. Consensus may increase the probability of—i.e., facilitate the process of reaching (or avoiding)—a particular future state of affairs, or increase the conviction that it will occur, but dissensus points up where issues are likely to arise, where incipient problems may lurk, where more information may be needed, or where the fact of diversity must be acknowledged and taken into account. In addition, it may stimulate synergistic thinking to resolve previously irresolvable differences in new creative ways. It is naturally interesting to relate diverse patterns of response on future images to independent variables describing individuals or groups [7].

Surprisingly, only limited research and debate have taken place on how indices of consensus can be influenced by *parameters of the Delphi survey itself*. The aim of this study was to understand the ways in which Delphi consensus measures are impacted by the Delphi survey characteristics. While there is a need to assess a broader range of consensus indices in the future, this study is the first report in the literature, to the best of our knowledge, that addresses the dependency of nine well-established consensus indices on the Delphi survey characteristics itself. As such, the present work is intended to stimulate debate and further research in science, technology and innovation management and strategic foresight communities.

## Materials and Methods

### Delphi study characteristics examined

Using a simulation approach, we evaluated the three key parameters whose variation conceivably can influence the observed consensus in Delphi studies:

1. number of survey questions varying from 6 to 40;
2. number of participating experts (i.e., the sample size) varying from 6 to 50; and
3. variation in the extent to which experts conform to group opinion (the Group Conformity Index, GCI) [8] in a Delphi study.

The GCI varied from 0.0 (the situation when an expert does not change her/his opinion in the course of the Delphi iterative rounds; the “stubborn/rigid” or the most opinionated expert) to 1.0 (when an expert is very likely to conform to group opinion; the “flexible/adaptable” or the least opinionated expert).

### Delphi consensus indices and their definitions

The impacts of variability in the above three Delphi characteristics on nine Delphi consensus indices were examined in 1000 simulations: Clustered Mode, Clustered Pairwise Agreement, Conger’s Kappa, De Moivre index, Extremities Version of the Clustered Pairwise Agreement, Fleiss’ Kappa, Mode, the Interquartile Range and Pairwise Agreement [9–12]. Their brief definitions are provided below.

- **De Moivre index** (*DM*) takes a value of 0 or 1. It determines whether all experts unanimously agree on a rating for a survey question.
- If *a*_*i*_ is the proportion of all pairs of experts in agreement over all possible pairs of experts for the survey question *I*, then **Pairwise Agreement** *PWA* is the corresponding average over all survey questions studied.
- *CPWA* is the **Clustered Pairwise Agreement**, i.e., the average over all questions of the proportions of pairs of experts in each consensus cluster over all possible pairs of experts.
- *XCPWA* is the **Extremities Version of the Clustered Pairwise Agreement.** It is similar to *CPWA*, but only looks at the frequency of ratings falling within either the lower or upper extreme ranges of points on the scale (e.g., 1-2-3 and 8-9-10 respectively in our simulation).
- *KF* and *KC* are **Fleiss’ Kappa** and **Conger’s Kappa** respectively. These “Kappa” multi-rater indices of consensus measure agreement between experts over that which would be expected by chance (although the way chance agreement is taken into account in the calculation of kappa indices has been criticized [10]). The different ways of calculating kappa differ mainly in the value of the probability that there is chance agreement, due to different assumptions of distributions of object ratings among all experts [11, 12].
- *M*, the **Mode**, is the proportion of experts who chose the score most popular in rating that object.
- *CM*, the **Clustered Mode**, calculates the proportion of experts who chose the cluster of scores most popular in rating the object.
- *IQR*, the **Interquartile Range**, is the measure of dispersion for the median, and consists of the middle 50% of the observations. Thus, to determine whether consensus has been achieved using this measure, a maximum threshold of *IQR* is established. If the middle 50% of observations range by less than the threshold, consensus is considered to have been achieved.

### Delphi simulations

We simulated a classic three-round Delphi survey building on the concept of clustered consensus/dissensus The Delphi simulation model was written in the multiplatform, open-source Python programming language.

In brief, for a given set of three Delphi survey characteristics—for example, 12 survey questions, 20 experts, and a GCI of 0.5 –a Delphi Round 3 rating score was obtained for each question under these conditions, using the uniform random distribution to generate Round 1 scores. Of note, in each Delphi Round 3, one obtains a value for a given consensus index for each question. In other words, if there are *N* number of questions in a Delphi survey, then one obtains *N* values for the consensus index in a Delphi survey. Hence, in each of our simulations, we averaged these final consensus index values across the questions to obtain a single study-wide consensus score. Then, this simulation process was repeated 1000 times yielding a rating score for each survey question under the above same survey conditions. The median value of the 1000 simulations of a study-wide consensus index was taken to represent these survey conditions (e.g., 12 survey questions, 20 experts, and a GCI value of 0.5).

The simulation process above was repeated to evaluate the impact of variation in one of the Delphi characteristics (e.g., the number of experts varying from 6 to 50) while the other two characteristics were kept constant. We used 12 survey questions, 20 experts, and a GCI value of 0.5 when one of these three Delphi characteristics varied to examine its impact on the consensus indices.

The dependency values displayed in Tables 1 to 3 represent the maximum difference (i.e., maximum median value minus minimum median value) observed when one of the Delphi characteristics varied. For example, in the case of the Delphi sample size (number of experts) varying from 6 to 50, if a dependency value of 0.300 is reported, this would mean that the maximum difference in the median values observed in each of the 1000 simulations carried out while varying the Delphi sample size condition from 6 to 50 was 0.300 for the given consensus index.

**Table 1:** RANK ORDER of the *Dependency* of Consensus Indices’ on the NUMBER OF QUESTIONS (6–40) in a Delphi Survey

Delphi Consensus Index	Dependence on the Number of Questions (0.000–1.000)*
Fleiss’ Kappa (KF)	.025
Conger’s Kappa (KC)	.019
Clustered Mode (CM)	.008
Extremity CPWA (XCPWA)	.005
Clustered PWA (CPWA)	.004
Mode (M)	.004
Pair-wise Agreement (PWA)	.002
De Moivre index (DM)	.000
Interquartile Range (IQR)	.000

*The dependency value ranges from 0.000 to 1.000. A value of “0.000” shows complete independence of the Consensus Index from the Delphi survey characteristic examined (e.g., the number of questions) whereas a value of “1.000” shows complete dependence. The dependency value is the maximum numeric difference observed for each consensus index when the number of questions in a simulated Delphi survey varied from 6 to 40.

All Delphi consensus indices (the *left column*) typically take a value ranging from 0.000 to 1.000, except the Interquartile Range (IQR). For example, in the case of Fleiss’ Kappa, a maximum difference of 0.025 can be anticipated when the number of Delphi survey questions vary from 6 to 40.

For the Interquartile Range, the dependency data were normalized by dividing the difference observed in simulations by the maximum possible difference (9.000), i.e., the length of the Likert scale from 1 to 10 used in the simulations.

**Table 2:** RANK ORDER of the *Dependency* of Consensus Indices’ on the NUMBER OF EXPERTS (Sample Size) (6–50) in a Delphi Survey

Delphi Consensus Index	Dependence on the Number of Experts in the Survey (0.000–1.000)*
Interquartile Range (IQR)	.396
Clustered Mode (CM)	.130
Mode (M)	.116
Clustered PWA (CPWA)	.072
Extremity CPWA (XCPWA)	.021
Fleiss’ Kappa (KF)	.021
Conger’s Kappa (KC)	.016
Pair-wise Agreement (PWA)	.015
De Moivre index (DM)	.000

*The dependency value ranges from 0.000 to 1.000. A value of “0.000” shows complete independence of the Consensus Index from the Delphi survey characteristic examined (e.g., the sample size) whereas a value of “1.000” shows complete dependence. The dependency value is the maximum numeric difference observed for each consensus index when the number of experts in a simulated Delphi survey varied from 6 to 50.

All Delphi consensus indices (the *left column*) typically take a value ranging from 0.000 to 1.000, except the Interquartile Range (IQR). For example, in the case of Clustered Mode, a maximum difference of 0.130 can be anticipated when the sample size varies from 6 to 50. For the IQR, the dependency data are normalized by dividing the difference observed in simulations by the maximum possible difference (9.000), i.e., the length of the Likert scale from 1 to 10 used in the simulations. Accordingly, the IQR can vary by a value of 0.396 when the Delphi sample size varies within the above range.

**Table 3:** RANK ORDER of the *Dependency* of Consensus Indices’ on the GROUP CONFORMITY INDEX in a Delphi Survey

Delphi Consensus Index	Dependence on the Group Conformity Index in the Survey (0.000–1.000)*
**Fleiss’ Kappa (KF)**	.504
**Conger’s Kappa (KC)**	.501
**Pair-wise Agreement (PWA)**	.480
**Mode (M)**	.429
**Clustered PWA (CPWA)**	.268
**De Moivre index (DM)**	.250
**Clustered Mode (CM)**	.200
**Extremity CPWA (XCPWA)**	.087
**Interquartile Range (IQR)**	.083

*The dependency value ranges from 0.000 to 1.000. A value of “0.000” shows complete independence of the Consensus Index from the Delphi survey characteristic examined (e.g., the Group Conformity Index) whereas a value of “1.000” shows complete dependence. The dependency value is the maximum numeric difference observed for each consensus index when the Group Conformity Index in a simulated Delphi survey varied from 0.0 to 1.0.

All Delphi consensus indices (the *left column*) typically take a value ranging from 0.000 to 1.000, except the Interquartile Range (IQR). For example, in the case of the Fleiss’ Kappa, a maximum difference of 0.504 can be anticipated when the Group Conformity Index varies from 0.0 to 1.0. For the IQR, the dependency data are normalized by dividing the difference observed in simulations by the maximum possible difference (9.000), i.e., the length of the Likert scale from 1 to 10 used in the simulations.

In addition to the above description of our simulations suitable for a general readership, we provide below a more technical description together with the attendant mathematical details.

### First-round simulation

In simulating the “experts” iteratively rating the survey questions on an ordinal scale, the first step was generating the Delphi first-round results using the discrete uniform random distributions.

### Clustered consensus and dissensus

In recent years, Delphi researchers have moved away from seeking consensus and towards utilizing the Delphi methodology to identify whether consensus exists and highlight, when it does not, areas of disagreement in order to then study the divergent views that may be just as important to govern innovations and emerging technologies [6, 13–15].

The reason to stress the importance of a possible lack of consensus—termed by some as “dissensus” [7, 13]–identified in a Delphi study is that there is a danger of stifling naturally occurring divergent views on issues and instead promoting an artificial consensus. Whether or not its value is recognized, dissensus does occur in Delphi studies. In the case of dissensus, a few opinion clusters containing the majority of experts can be identified. These clusters indicate the communities holding divergent views. Indeed, dissensus, as the flipside of consensus, may be termed clustered consensus. Modifying the traditional definition of consensus to allow for clusters, it may be generalized that all consensus is clustered, where there may theoretically exist between one and infinity of clusters, but where in practice there are at most only a few of significant size.

### Cluster identification and convergence

Our simulation of later (2^nd^ and 3^rd^) rounds of a Delphi study took into consideration the concept of consensus clusters, where simulated “experts” adjusted their previous round’s rating for each survey question approaching the mean rating of a consensus cluster identified in the previous round for the question. This allowed for a realistic between-round dynamic in cases of group dissensus.

After generating first-round scores by the discrete uniform random distribution, the next step was establishing the cluster size (***v***) upon which the significant clusters of experts can be identified. This value (***v***) depends on the researcher’s needs. In our simulations, clusters comprising three points on a 1 to 10-point scale were chosen (i.e., a 9-point Likert scale); this is similar to that recommended by the RAND online resource [16].

There are many algorithms to choose from when identifying the consensus cluster(s). In the present study, the mode was determined, i.e., the cluster most raters’ scores fell within. The number of ratings falling within the mode is thus *r*_*mode*_. Second, a threshold *p* (0<*p*<1) was decided upon, such that if some other interval (of maximum size *v*) contained at least p*r_*mode*_ ratings, it would have constituted another cluster towards which a portion of the experts would have converged in the following round.

To state that experts converge towards a cluster in the following round means that experts converge towards a measure of the central tendency of that cluster, such as the mean, median or mode. In our simulation, we used the mean. When more than one cluster was identified, the expert converged towards the cluster nearest to her/his recent rating, or in the case of being exactly midway—towards the larger of the two. It is not always necessary to simulate experts converging to the nearest cluster; indeed, it is possible to observe experts changing their opinion radically in real-world Delphi studies. In order to prevent outlying smaller clusters from pulling in too many adjacent experts and leaving a more popular but “isolated” cluster neglected, which could possibly occur in certain conditions with an excessively low threshold *p*, it is suggested that the value of *p* be chosen as greater than or equal to 0.5.

Convergence in the simulations used the Group Conformity Index (GCI) (or what has been alternatively called conformity index (*β)* [8]). More specifically, a rater *j* that scored an item *k* in round *t* will in the next round *t* ^*+1*^ score it as, rounded to the nearest possible value,

$$\left(CGI*mean_{j}+\left(1-CGI\right)*k\right)$$

where *mean*_*j*_ is the mean of the cluster that the rater *j* converges towards in round *t* ^*+1*^ as described above. A change in the CGI used in a simulation should ideally be reflected by a corresponding change in the consensus index reported. Further research may also investigate, we suggest, fuzzy conformity indices, where each expert’s CGI could come from a specified range of values, thus modeling individuals’ differing levels of conformity or “stubbornness” [17].

The procedure of identifying clusters in round *t* and converging towards them in round *t* ^*+1*^ is the same regardless of the value of *t*. The simulations were run for three rounds, which was sufficient to observe the behaviour of the different consensus indices and consistent with contemporary empirical Delphi studies [18–20].

### Simulation of variations

Each simulation was run 1000 times. Each time, consensus indices for each survey question as well as the aggregate study-wide consensus index value were calculated. Thereafter, simulations were run for the variations of the Delphi characteristics, varying one at a time the following parameters: the conformity index (between 0.0 and 1.0), the number of experts (between 6 and 50), and the number of survey questions (between 6 and 40).

### Formulae for calculating consensus indices

There are a total of *n* items, numbered i = 1…n; there are *q* possible ratings, numbered j = 1…q; and there are *r* experts, numbered k = 1…r.

$$DM=\frac{\sum_{i=1}^{n}c_{i}}{n}$$

$$c_{i}=\{\begin{array}{cc} 1 & if\;\sum_{k^{\prime }>k}\;c_{ikk^{\prime }}=r\left(r-1\right)/2\\ 0 & otherwise \end{array}$$

is the item-by-item *DM*

$$c_{ikk^{\prime }}=\{\begin{array}{cc} 1 & if\;q_{ik}=q_{ik^{\prime }}\\ 0 & otherwise \end{array}$$

*q*_*ik*_ is the rating given to object *i* by expert *k* *C*_*ikk'*_ is 1 if experts *k* and *k’* agree on object *i* and 0 if they do not

$$PWA=\frac{\sum_{i=1}^{n}a_{i}}{n}$$

$$a_{i}=\frac{\sum_{k^{\prime }>k}c_{ikk^{\prime }}}{r\left(r-1\right)/2}$$

where *a*_*i*_ is the item-by-item *PWA*.

$$CPWA=\frac{\sum_{i=1}^{n}ac_{i}}{n}$$

$$ac_{i}=\overset{q}{\underset{j=1}{\sum }}\frac{\left(\sum_{k=1}^{r}d_{ikj})(\sum_{k=1}^{r}d_{ikj}-1\right)}{r\left(r-1\right)}$$

where *ac*_*i*_ is the item-by-item *CPWA* and

$$d_{ikj}=\{\begin{array}{cc} 1 & if\;q_{ik}\in cluster_{j}\\ 0 & otherwise \end{array}$$

*cluster*_*j*_ is a consensus cluster

$$XCPWA=\frac{\sum_{i=1}^{n}acx_{i}}{n}$$

$$acx_{i}=\frac{\left(\sum_{k=1}^{r}d_{ikl})(\sum_{k=1}^{r}d_{ikl}-1)+(\sum_{k=1}^{r}d_{iku})(\sum_{k=1}^{r}d_{iku}-1\right)}{r\left(r-1\right)}$$

where *acx*_*i*_ is the item-by-item *XCPWA* and

$$d_{ikl}=\{\begin{array}{cc} 1 & if\;q_{ik}\in group_{l}\\ 0 & \;otherwise \end{array}$$

$$d_{iku}=\{\begin{array}{cc} 1 & if\;q_{ik}\in group_{u}\\ 0 & otherwise \end{array}$$

*group*_*l*_ are the extreme lower bound ratings *group*_*u*_ are the extreme upper bound ratings

$$KF=\frac{P-P_{e,k}}{1-P_{e,k}}$$

$$P=\frac{\sum_{i=1}^{n}\sum_{j=1}^{q}r_{ij}^{2}-nr}{nr\left(r-1\right)}$$

*r*_*ij*_ is the number of experts giving rating *j* to object *i*

$$P_{e,k}=\sum_{j=1}^{q}{\left(\sum_{i=1}^{n}r_{ij}/nr\right)}^{2}$$

*r*_*ij*_ is the number of experts selecting rating *j* for question *i*

$$KC=\frac{P-P_{e,C}}{1-P_{e,C}}$$

$$P_{e,C}=P_{e,k}-\sum_{j=1}^{q}s_{jk}^{2}/\left(r-1\right)$$

$$s_{jk}^{2}=\frac{\left[r\sum_{k=1}^{r}{\left(n_{jk}\right)}^{2}-{\left(\sum_{k=1}^{r}n_{jk}\right)}^{2}\right]}{n^{2}r^{2}}$$

*n*_*jk*_ is the number of items expert *k* has rated *j*

$$M=\frac{\sum_{i=1}^{n}s_{i}}{n}$$

$$s_{i}=\frac{\sum_{k=1}^{r}s_{ik}}{r}$$

is the item-by-item *M*

$$s_{ik}=\{\begin{array}{cc} 1 & if\;q_{ik}=mode_{i}\\ 0 & otherwise \end{array}$$

*mode*_*i*_ is the rating given to object *i* by the biggest number of experts

$$CM=\frac{\sum_{i=1}^{n}cs_{i}}{n}$$

$$cs_{i}=\frac{\sum_{k=1}^{r}cs_{ik}}{r}$$

is the item-by-item C*M*

$$cs_{ik}=\{\begin{array}{cc} 1 & if\;q_{ik}\in cl.mode_{i}\\ 0 & otherwise \end{array}$$

*cl*.*mode*_*i*_ is the cluster most raters’ scores fell within The item-by-item *IQR*_*i*_ *= q*_*75*,*i*_ *-q*_*25*,*i*_ *q*_*m*,*i*_ is the rating below which *m*% of all ratings for object *i* fall

$$IQR=\frac{\sum_{i=1}^{n}IQR_{i}}{n}$$

## Results

We present the *rank order* of the dependency of the nine commonly used Delphi consensus indices on variations in three salient Delphi survey characteristics, the number of survey questions, the sample size and the Group Conformity Index, in Tables 1–3.

The number of questions (range: 6 to 40) in a survey did not have a notable impact whereby the dependency values remained beyond the first decimal point 0.030 (Table 1). The variation in sample size (range: 6 to 50) displayed the top three impacts for the Interquartile Range, the Clustered Mode and the Mode (dependency = 0.396, 0.130, 0.116, respectively) (Table 2). On the other hand, the Group Conformity Index greatly impacted all nine Delphi consensus indices (dependency = 0.200 to 0.504), except the Extremity CPWA and the Interquartile Range that were impacted only beyond the first decimal point (dependency = 0.087 and 0.083, respectively) (Table 3).

## Discussion

Emerging technologies and knowledge-based innovation often face a volatile development trajectory. Some discoveries dissipate in obscurity while others become full-fledged innovative products, adopted in society, markets and medical practices worldwide. Even a small steering shift made *early* in the development course of a highly novel technology and innovation can, therefore, accrue important momentum and weight in the course of time, and as innovations diffuse into various geographies and socio-technical application contexts. Hence, there is an increasing tendency for *anticipatory* governance of new technologies and innovations while social and scientific change are both possible [21]. It is in this particular context that Delphi studies are increasingly being utilized to inform technology foresight and multiple future innovation scenarios [6].

Despite their growing popularity, there is little guidance available on Delphi design, implementation, reporting or interpretation, with the notable exception of the works by Sinha and colleagues [5]. These authors have recommended a thorough and systematic checklist to be reported in studies using the Delphi technique, in a context of determining consensus on which outcomes ought to be measured in clinical trials or systematic reviews [5]. Yet, there is no former research, to the best of our knowledge, that examined the ways in which variations in the salient aspects of the Delphi design, namely, the number of questions posed to the survey participants, the number of experts (sample size) in the survey and the Group Conformity Index, impact the observed consensus in a Delphi survey, and how different consensus indices might have differential sensitivities to these key Delphi characteristics.

Our results show that the number of questions in a Delphi survey, when they vary from 6 to 40, do not appreciably impact the nine frequently used consensus indices, and appear to change the consensus values only in the second decimal level (Table 1). This range of questions represents a typical survey question volume: Delphi participants are experts (scientists, policymakers, CEOs, community leaders, etc.) typically with busy work schedules and are unlikely to dedicate a lengthy time, usually no more than 30 to 45 minutes, for the survey. Most Delphi research designers consider this reality—that the respondents are time constrained experts—and thus, plan for surveys with question contents that can be reasonably completed under an hour to secure in-depth answers from the respondents.

Delphi surveys are a form of qualitative research that generate hypothesis (rather than quantitative hypothesis testing), and can help uncover social dimensions of science and technology hitherto underexplored or silenced due to power and equity differences in society. We note that the literature on Delphi surveys traditionally recommends a panel of 10 to 15 experts, typical of most qualitative research [4, 20]. While there are Delphi surveys with a large sample size in the order of a few hundreds [22], they tend to embody the additional purpose of hypothesis testing or confirmation of respondents’ opinion. The present study informs the typical qualitative research and hypothesis generation oriented Delphi surveys with a sample size range from 6 to 50.

The Group Conformity Index ranges from 0.0 to 1.0. It is a construct akin to measuring stubbornness (GCI = 0.0) and flexibility (GCI = 1.0) of experts’ opinions. GCI is critical in gauging the malleability of experts’ opinions in contested knowledge domains such as emerging technologies and innovations. The Delphi simulations in the present study employed a GCI range from 0.0 (most opinionated “stubborn” expert) to 1.0 (least opinionated “flexible/adaptable” expert).

We underscore in this context that such qualities are not only dependent on the individual agency (freewill or personality) of an expert but also the larger social and political innovation climate, values and agendas in which such innovation actors (e.g. scientists, policymakers, funding agencies) are embedded [23–32]. Consider, for example, the case of a highly contested emerging technology facing a highly volatile innovation trajectory due to local and global political, economic and societal stakes involved. These can be technologies impacting, for example, stem cell research and aging, military and defense industries or technologies involved in renewable energy in the face of a rapidly aging and energy-hungry planet. Similarly, innovation actors in autocratic states may also be under pressure to conform to certain local political conjectures beyond their own agency/freewill or independent choice. Experts in a Delphi survey may thus be under influence to conform and entrench in their local milieu (thus creating personal blind spots and compromised objectivity) due to external sociopolitical pressures [23]. Our simulation study suggests that in such highly volatile social and political contexts the Delphi researcher may want to take into consideration the dependence of experts’ opinion on variation in GCI when deciding on which consensus index to use.

The simulation results contextualized above offer constructive ways forward in selecting the Delphi consensus indices to be used based on the Delphi characteristics (Tables 1–3). They also help interpret the reported Delphi study findings in the literature that have been conducted without adequate attention to variations in these Delphi survey characteristics.

We re-emphasize that a simulation approach was used to examine the dependencies of the mainstay consensus indices on the Delphi survey characteristics. In terms of statistical cutoffs such as p-values or a normative threshold to declare dependency, we believe a rank order of dependencies is more robust. In simulations, it is generally believed that statistical hypothesis tests are not appropriate or misleading because p-values are determined by statistical power (i.e., replication) [33], which can be artificially high in a context of simulations, producing minuscule p-values if and when desired [34]. We therefore suggest that modeling and simulation studies can be misleading by focusing on p-values and that presenting rank order of dependencies as displayed in this report appropriately inform the readership in a context of Delphi design, and/or interpretations of consensus claims in the future. The rank order data presented in Tables 1 to 3 show that the Delphi characteristics, particularly the number of experts in a Delphi survey and the Group Conformity Index influence the numeric values of consensus observed in such foresight research.

There are several potential shortcomings of the present simulation study. First, there is debate and uncertainty regarding the calculation of kappa measures as a consensus measure [35, 36]. Second, in an empirical Delphi study with non-simulated experts, qualitative between-round feedback plays an important role in a respondent’s decision whether or not to change her or his rating. In a simulated environment, however, it is not possible to non-randomly model such behaviour. Thus, all shifts of opinion (or lack thereof) are simulated using numerical data exclusively.

## Conclusions and Future Ramifications

While the Delphi research is extensively utilized in assessment of the emerging fields of medical and life sciences innovation in regards to presence of consensus among expert communities, surprisingly, little discussion has taken place on the factors that can influence the conclusions drawn from Delphi surveys. *PLOS ONE* publishes a wide range of Delphi research articles on technology foresight and hence, the findings reported herein might help future researchers and the readers of the journal better interpret Delphi findings and importantly, choose the appropriate consensus measure indices depending on their anticipated survey characteristics.

Additionally, technology foresight and implementation actors such as policymakers, governments, academics, technology entrepreneurs and scholars involved in foresight development and future(s) studies would be informed by the findings presented in this study [26, 37–39].

This study has additional salient implications for future research on the Delphi technique using computer simulations. Delphi simulations can be used for understanding the dynamics behind observable behaviour of Delphi data, for probing the cause and effect relationships between different Delphi characteristics, and foresight/implementation science related outcomes such as consensus, dissensus, conflict or synergy among experts in an innovation ecosystem. Further approaches to analyzing and contextualizing Delphi design, data and findings, including using computer simulations and clusters of consensus, are recommended.
